# Uncovering Important Drivers of the Increase in the Use of Virtual Care Technologies in Nursing Care: Quantitative Analysis From the 2020 National Survey of Canadian Nurses

**DOI:** 10.2196/33586

**Published:** 2022-03-31

**Authors:** Waldo Beauséjour, Simon Hagens

**Affiliations:** 1 Canada Health Infoway Toronto, ON Canada

**Keywords:** adoption of virtual care, secure messaging, nurses, nursing, telehealth, telehomecare, telemonitoring, remote patient monitoring, virtual videoconferencing, uptake of virtual care

## Abstract

**Background:**

Canadian nurses are at the forefront of patient care delivery. Although the use of digital health technologies for care delivery is gaining momentum in Canada, nurses are encouraged to integrate virtual care into their practice. In early 2020, more Canadian nurses delivered care virtually compared with 3 years ago.

**Objective:**

This study seeks to uncover the professional characteristics of Canadian nurses accessing virtual care in 2020, understand how these characteristics differ across types of technologies, investigate whether the nurses accessing virtual care possess the skills and knowledge needed to use these technologies, and determine the important drivers of the uptake of virtual care observed in 2020.

**Methods:**

We used data from the 2017 and 2020 National Survey of Canadian Nurses. This survey collected data on the use of digital health technologies in nursing practice. It concerned regulated nursing professionals working in different health care settings and from different domains of nursing practice. We combined the chi-square independence test and logistic regression analysis to uncover the most relevant drivers of virtual care uptake by nurses in 2020.

**Results:**

In early 2020, before the declaration of the COVID-19 pandemic, nurses who delivered care virtually were predominantly nurse practitioners (135/159, 84.9%) and more likely to work in a primary or community care setting (202/367, 55%) and in an urban setting (194/313, 61.9%). Factors such as nursing designation (*P*<.001), perceived quality of care at the health facility where the nurses practiced (*P*<.001), and the type of patient record–keeping system they had access to (*P*=.04) had a statistically significant effect on the probability of nurses to deliver care virtually in early 2020. Furthermore, nurses’ perception of the quality of care they delivered through virtual technologies was statistically associated with their perception of the skills (*χ^2^*_4_=308.7; *P*<.001) and knowledge (*χ^2^*_4_=283.4; *P*<.001) to use these technologies.

**Conclusions:**

This study emphasizes the critical importance of nursing designation, geographic location, and type of patient record–keeping system in predicting virtual care integration in nursing practice. The findings related to geographic location can be used by decision-makers for better allocation of digital health resources among care settings in rural and urban areas. Similarly, the disparities observed across nursing designations have some implications for the digital training of nurses at all levels of practice. Finally, the association between electronic medical record use and uptake of virtual care could accelerate the implementation of more modernized record-keeping systems in care settings. Hence, this could advance interoperability and improve health care delivery.

## Introduction

### Background

Whether it is a hospital, nursing home, or community-based health facility, most Canadian health care settings have shifted to virtual care during the COVID-19 pandemic. However, before the pandemic, the adoption of this model of care delivery was already trending upward in Canada [1]. As nurses account for a large proportion of the workforce in these care settings and are at the forefront of patient care delivery, they are well positioned to benefit from the integration of virtual care technologies into clinical practice.

Virtual care refers to “any interaction between patients and members of their circle of care, occurring remotely, using any forms of communication or information technologies, with the aim of facilitating or maximizing the quality and effectiveness of patient care” [2]. It can be regarded as a multimodal technology that embodies telephone, videoconferencing, and messaging. In addition, virtual care can be viewed as a multipurpose technology for (1) remote patient monitoring (RPM), (2) telehealth, (3) patient education, (4) peer-to-peer education, and (5) health information exchange between patients and providers. Some authors have described virtual care as a mature version of telehealth [3,4]. As the Canadian health care system is increasingly embracing digital health solutions, more clinicians and patients are expected to use virtual care technologies. In Canada, the proportion of nurses virtually delivering care has increased substantially from 20% to 51% between 2017 and 2020 [5]. Although this growth was observed a few weeks before the pandemic, the progressive integration of virtual care technologies into nursing practice is very promising. These technologies can potentially augment nurses’ capability to deliver care in nontraditional ways and improve their scope of practice [6]. Nevertheless, to sustain this progress in a changing health care landscape, it is crucial to understand the promotors and inhibitors of the uptake of virtual care technologies by nurses.

Our review of the existing literature mainly focused on identifying potential enablers of and barriers to virtual care adoption in nursing practice. Our brief search demonstrated that three sets of enabling and inhibiting factors had been studied by previous researchers: (1) personal and professional characteristics of the nurses, (2) factors related to the care setting where the nurses practice, and (3) factors related to the patients they serve. For the first set of factors, numerous researchers have highlighted training in virtual care technologies as one of the key ingredients that can enhance the technological skills of nurses [7-10]. This professional enhancement can consequently create a sense of confidence, which in turn could lead to the adoption of digital technologies in nursing care [11]. Nonetheless, if the training was only technology focused and neglected clinical and practice aspects, it could impede adoption by frontline staff [7]. In addition, enhancing nurses’ skills through training should be accompanied by ongoing technical support [12].

Nurses’ attitudes toward the adoption of virtual care were also one of the personal factors explored by previous studies. Nurses with positive previous experiences with other digital health technologies such as electronic medical records (EMRs) were more inclined to integrate virtual care in their practice [12,13]. Fronczek et al [14] believed that providing nurses with additional education and experience during their career would equip them with the skills required to practice in virtual care environments.

Regarding care setting–related factors, previous studies have mostly emphasized the following as the main enablers of and barriers to the uptake of virtual care by nurses: (1) policy [7,15], (2) good collaboration between services [7,12], (3) changing working environment [7], (4) lack of encouragement from managers [9], (5) lack of coordination of services [12], (6) ambiguity around the objective of integrating virtual care technologies into clinical practice [7], and (7) existence of written guidance on the use of these technologies [12]. Among these factors, policy seemed to be an important element. Garber and Chike-Harris [15] maintained that it is essential for nurse practitioners (NPs) to be knowledgeable about the policies affecting their practice when delivering care through telehealth.

Enablers and barriers pertaining to the technology per se involved five factors [9,12,16]: (1) ease of use, (2) flexibility, (3) contextual integration of the technology, (4) privacy, and (5) security. For privacy and security, both nurses and patients must be assured that they can use virtual care technologies safely [6]. However, Hardcastle and Ogbogu [17] clearly stated, “ensuring data security is arguably the most challenging aspect of virtual care.”

Most of the previous studies cited above [7-16] concentrated on identifying the factors driving the integration of virtual care into nursing practice. They also focused on finding out whether these factors affected nurses’ adoption positively or negatively. There is a paucity of research investigating the weight of each of these factors in predicting the adoption of virtual care technologies in nursing practice. Therefore, it is almost difficult to decide which factors to prioritize or which one would have the greatest impact on adoption from a policy standpoint. In addition, these previous studies [9,10,13,16] were mostly systematic reviews aiming to inventory the different enablers of and barriers to the adoption of virtual care technologies by nurses. They used qualitative research methods, including (1) focus group discussions, (2) in-depth or semistructured interviews, and (3) ethnographic methods. Hence, the small sizes along with the nonprobabilistic nature of the samples used in these studies restricted their ability to infer their results to a larger nursing workforce. In addition, some previous findings concerned specific forms of virtual care technologies, notably videoconferencing and RPM, or focused on particular care settings. Building on these previous findings, our research contributes to filling the quantitative analysis gap in the literature on the drivers of virtual care adoption by nurses. In addition to quantifying the joint influence of the factors of adoption discussed above, our empirical research attempts to estimate the magnitude of the impact of each of these factors on the uptake of virtual care by nurses.

### Objectives and Research Questions

The objective of this paper is twofold: (1) to examine the characteristics of Canadian nurses using virtual care technologies and (2) to investigate the drivers of the uptake of these technologies by nurses in direct patient care in 2020. Mapping the profile of nurses accessing virtual care technologies should help better address barriers to accessing these technologies. It should also help determine whether nurses have access to appropriate digital health solutions to enhance their scope of practice. More specifically, our study seeks to (1) uncover the professional characteristics of Canadian nurses accessing virtual care technologies in 2020; (2) understand how these characteristics differ across types of technologies, geographic location, and care settings; (3) investigate whether nurses accessing virtual care possess the skills and knowledge they need to use these technologies; and (4) determine the important drivers of the uptake of virtual care observed in 2020.

## Methods

### Data Source

The analysis used data generated from the 2020 National Survey of Canadian Nurses. This survey was designed to be representative of the entire Canadian nursing workforce, particularly nurses in direct patient care. The survey is conducted on a triennial basis. Two iterations of the survey were conducted in 2014 and 2017. The National Survey of Canadian Nurses mainly gathers data on the use of digital health technologies in nursing practice, more specifically, the use of EMR systems and virtual care technologies.

### Study Population and Sample

The population of reference of the National Survey of Canadian Nurses comprised regulated nursing professionals working in different health care settings and from different domains of nursing practice, with a focus on nurses in direct patient care. These nursing professionals comprised (1) registered nurses (RNs), (2) NPs, (3) clinical nurse specialists (CNSs), (4) licensed practical nurses (LPNs), and (5) registered psychiatric nurses (RPNs). This categorization of regulated nursing professionals aligns with the definition adopted by the Canadian Institute for Health Information (CIHI) [18], except that the National Survey of Canadian Nurses also considered another group of regulated nursing professionals, called CNS. In 2019, the total number of regulated nurses in Canada was 439,975 [18].

During the design phase of the National Survey of Canadian Nurses, the survey confronted the lack of a pre-existing sampling frame that would have provided a complete list of all the regulated nursing professionals listed above. To construct an alternative sampling frame, the survey conflated the membership lists provided by the Canadian Nurses Association (CNA) and Canadian Nursing Informatics Association (CNIA). We recognize that the use of this combined membership list as an alternative sampling frame may have coverage problems, meaning that all the participants from the population of interest may not be included in the survey frame. Nevertheless, both the CNA and CNIA have a large network of nurses, which accounts for a substantial proportion of the Canadian nursing workforce. Hence, their membership lists guaranteed an acceptable level of coverage for the 2020 National Survey of Canadian Nurses. In addition, to mitigate the lack of coverage and completeness of the sampling frame created from the combination of the CNA and CNIA membership lists, several strategies were implemented. They included (1) sending invitation emails to members of the l’Ordre des Infirmières et Infirmiers du Québec, (2) recruiting nurses from a web panel owned by the firm commissioned to field the survey, and (3) applying snowball methods by sending invitation emails to nurses referred by other nurses who had responded to the survey [5]. A total sample size of 1642 nurses was collected across Canada, 1132 (68.94%) of whom provided direct patient care. The National Survey of Canadian Nurses used a nonprobability sample; therefore, a margin of error cannot be associated with the sample.

### Data Collection Instrument and Procedures

The 2020 National Survey of Canadian Nurses used a bilingual (English and French), user-friendly, and 30-minute-long web-based questionnaire. This questionnaire asked questions on EMR systems and virtual care technologies used by nurses providing direct patient care in their main care settings. In addition, several demographic data were collected during the survey: (1) primary domain of nursing practice, (2) nursing designation, (3) primary care setting, and (4) geographic location of the care setting where the nurse practiced. The survey link was distributed to all nurses from the CNA and CNIA membership lists through email invitations between January 20 and March 29, 2020. To boost the survey response rate, a multipronged modified Dillman [19] approach was implemented, including an incentive strategy. In addition, ethics approval was obtained before fielding the survey. The ethics approval was granted by an independent Canadian institutional review board in January 2020 (Advarra Inc; approval number: Pro00041060). It concerned the survey methodology or protocol, informed consent document, and data collection instrument. All the results expounded in this study were statistically weighted by the 2018 CIHI workforce data published in 2019 to ensure that they accurately represented the Canadian nursing workforce. A full description of the sample of Canadian nurses achieved in 2020 is provided in the survey report [5]. Similarly, a full version of the 2020 survey questionnaire along with the raw survey data can be accessed via the University of Victoria Dataverse portal [20].

### Statistical Analysis

The data used in this paper concerned point-of-care nurses using virtual care technologies to deliver care in their main setting from the 2020 National Survey of Canadian Nurses. Data on 4 types of virtual care technologies were collected in 2020. The working definitions for these technologies are provided below (questions on access to virtual care technologies that were used in the National Survey of Canadian Nurses are provided in Multimedia Appendix 1):

1. Secure messaging or email refers to consultations via secure email or SMS text messages sent by patients to their health care provider (in this case, the nurses) about a specific health question or concern.
2. Videoconferencing refers to virtual visits conducted between patients while at home and their health care provider via face-to-face web-based virtual encounters. These virtual visits are more likely to be patient initiated.
3. Telehealth is a form of virtual care delivered through videoconferencing that is coordinated and facilitated by or between health facilities. It involves a remote clinician, who is the main provider, aided by another clinician based in the rural or remote region where the patient receiving the care resides. The patient can be either at home or at a health facility when receiving care [9].
4. RPM or telehome care occurs when a health provider (typically a nurse or paramedic) has electronic access to a patient’s information for review, interpretation, and coaching opportunities to enhance the patient’s self-management abilities.

Previous researchers [21-23] have favored the term information and communication technologies to refer to all digital technologies used to deliver care remotely. This delineation embodies a large palette of digital health technologies, including EMRs or electronic health record systems, which aim more at dealing with patients’ health information or supporting clinical administrative activities [13]. This description seemed to be overly broad for the scope of our analysis as our definition of virtual care technology is not inclusive of EMRs and electronic health records. Instead, we limited our definition to the 4 aforementioned virtual care technologies collected from the 2020 National Survey of Canadian Nurses. In addition, our study used the terms virtual care use, uptake, and adoption interchangeably. Chen et al [24] relied on the number of times a telehealth technology was used to measure telehealth adoption. Their use index was quantitative in nature. Unlike these scholars, we defined binary use variables for the 4 virtual care technologies studied. For our study, *use* referred to the use of a virtual care technology by a nurse at least once, whereas a variable referred to a characteristic observed or collected for the nurses during the survey. Moreover, we used variable and factor interchangeably throughout the paper. We define our binary use variables as follows:



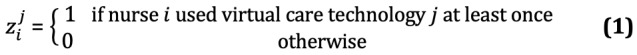



Here, the subscript *i* denotes the nurse, whereas *j* refers to the 4 virtual care technologies considered. The subscript *j* took the following values:



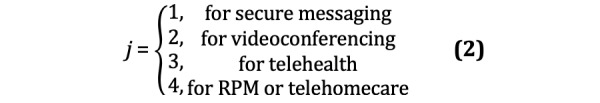



By conflating these 4 use factors, we created another binary use variable *y_i_*. This combined variable refers to the use of at least one of the 4 virtual care technologies; thus, it is the virtual care adoption variable. It constituted the main dependent variable of our analysis and was defined by the following equation:



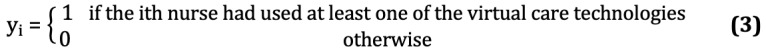



The explanatory variables were derived from the body of literature on the adoption of virtual care by nurses, which has been expounded previously. These previous studies predominantly emphasized two groups of factors influencing the use of virtual care technologies in nursing care: nurses’ skills and attitudes and factors pertaining to care settings where the nurses practice. On the basis of this, we retained the following independent variables: (1) nursing designation, (2) work experience (number of years working as a nurse), (3) skills and knowledge to use virtual care technologies, (4) perception of the quality of care delivered by the care setting where the nurse practices, (5) type of care setting, (6) geographic location of the setting, (7) patient record–keeping system used in the main care setting, and (8) policy about the use of virtual care technologies. These characteristics were analyzed using summary descriptive statistics and frequency tables. Moreover, the chi-square independence test along with association measures was used to test for the association between the use variables *z^j^_i_* and the independent factors retained. The joint effect of these predictors and their respective effects on virtual care adoption *y_i_* were estimated through the specification of the following logistic regression model:



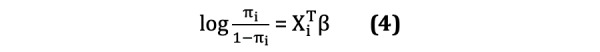



where *X_i_* is a vector of observed explanatory variables representing the characteristics defined above for nurses, whereas *β* refers to a vector of regression coefficients, and *π_i_* represents the probability that nurse *i* uses at least one virtual care technology. It is linked to the use variable *y_i_* through the following equation:



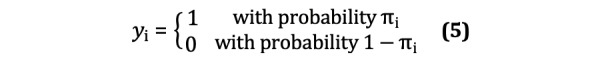



## Results

### Current State of Virtual Care Use by Canadian Nurses

Table 1 shows the trends in the adoption of virtual care by Canadian nurses for each technology considered in the analysis between 2017 and 2020. This shows that the proportion of nurses virtually delivering care increased substantially between 2017 and 2020, with an overall average increase of 25 percentage points for the 4 technologies considered. The greatest rise was observed for secure email (+27 percentage points), whereas the smallest increase was noted for telehealth, indicating a more rapid uptake of the former and a slow adoption for the latter. Admittedly, some of these technologies require more infrastructure and technological resources than others do for their rollout in care settings. Data from the 2020 National Survey of Canadian Nurses were collected a few weeks before the World Health Organization declared COVID-19 a global pandemic; therefore, one may argue that the COVID-19 pandemic may not be the main driver of the surge in the use of virtual technologies by nurses observed in early 2020. Nevertheless, this progressive virtualization of nursing practice could help the Canadian health care system cope with the unprecedented increase in the demand for digital care by patients and streamline the deployment of nurses as they are at the frontline of the response to the pandemic [25]. In addition, it is predicted that Canadian patients are likely to seek care through virtual modalities beyond the pandemic [26], thereby pushing nursing practice to adapt to these virtual care environments moving forward.

**Table 1:** Access to virtual care technologies by Canadian nurses in 2017 (N=1342) and 2020 (N=1047)^a^.

Virtual care technologies	Value		
	2017, n (%)	2020, n (%)	Percentage point change^b^
Secure message	121 (9.02)	377 (36.01)	27
Videoconference	40 (2.98)	283 (27.03)	24
Telehealth	81 (6.04)	304 (29.04)	23
RPM^c^	107 (7.97)	356 (34)	26
At least 1 of the 4 technologies	268 (19.97)	534 (51)	31

^a^Source: 2017 and 2020 National Survey of Canadian Nurses, Canada Health Infoway, Environics, and Léger [5,27].

^b^Percentage point change refers to the difference between the percentage of nurses accessing virtual care technology in 2020 and the proportion accessing the same technology in 2017.

^c^RPM: remote patient monitoring.

### Main Characteristics of Nurses Who Used Virtual Care Technologies in 2020

#### Overview

Tables 2 and 3 show the number and proportion of nurses who delivered care virtually in 2020 according to the type of technology. The results in Table 2 are presented for the personal and professional characteristics considered in the analysis, notably age, work experience, nursing designation, and skills and knowledge in delivering care through videoconferencing and telemonitoring. The results in Table 3 pertain to the characteristics of the main care settings where the nurses surveyed practiced, notably the geographic location of the care setting, the type of setting, and the patient record**–**keeping system used at the care setting.

**Table 2:** Personal and professional characteristics of Canadian nurses who used virtual care (VC) technologies in 2020 (N=1047)^a^.

Factors or covariates		VC technologies					
		Secure email	Videoconferencing	Telehealth	RPM^b^	At least one VC technology	Did not use any VC technology
Age (years), mean (SD)		40.6 (11.7)	39.6 (11.2)	38.7 (10.4)	39.5 (11.0)	41.9 (12.3)	42.9 (11.9)
Work experience (years), mean (SD)		13.4 (11.1)	12.3 (10.8)	11.5 (10.3)	12.8 (11.7)	15.3 (12.3)	16.7 (11.8)
**Nursing designation, n (%)**							
	RN^c^ (n=683)	171 (25)	116 (17)	137 (20.1)	164 (24)	280 (41)	403 (59)
	NP^d^ (n=159)	122 (76.7)	114 (71.7)	114 (71.7)	119 (74.8)	135 (84.9)	24 (15.1)
	LPN^e^, RPN^f^, and CNS^g^ (n=143)	63 (44.1)	40 (28)	44 (30.8)	60 (42)	87 (60.8)	56 (39.2)
	Other (n=61)	22 (36.1)	13 (21.3)	11 (18)	17 (27.9)	28 (45.9)	32 (52.5)
**Have the skills to use videoconference or telemonitoring, n (%), n=407**							
	Strongly and moderately agree	N/A^h^	232 (57)	N/A	232 (57)	N/A	N/A
**Have the knowledge to use videoconference or telemonitoring, n (%), n=407**							
	Strongly and moderately agree	N/A	243 (59.71)	N/A	243 (60)	N/A	N/A

^a^Source: 2020 National Survey of Canadian Nurses, Canada Health Infoway, and Léger [5,27].

^b^RPM: remote patient monitoring.

^c^RN: registered nurse.

^d^NP: nurse practitioner.

^e^LPN: licensed practical nurse.

^f^RPN: registered psychiatric nurse.

^g^CNS: clinical nurse specialist.

^h^N/A: not applicable.

**Table 3:** Characteristics of the main care settings where the Canadian nurses accessing virtual care (VC) technologies in 2020 practiced (n=1047)^a^.

Factors		VC technologies, n (%)					
		Secure email	Videoconferencing	Telehealth	RPM^b^	At least one VC technology	Did not use any VC technology
**Perception of overall quality of care at care setting**							
	Poor (n=97)	25 (26)	6 (6)	15 (15)	8 (8)	36 (37)	61 (63)
	Fair (n=181)	45 (24.9)	18 (9.9)	25 (13.8)	25 (13.8)	60 (33.1)	121 (66.9)
	Good (n=425)	183 (43.1)	145 (34.1)	149 (35.1)	187 (44)	242 (56.9)	183 (43.1)
	Excellent (n=344)	124 (36)	110 (32)	114 (33.1)	138 (40.1)	193 (56.1)	151 (43.9)
**I provide more efficient health care with virtual videoconferencing or telemonitoring (n=407)**							
	Strongly and moderately agree	N/A^c^	195 (47.91)	N/A	195 (47.91)	N/A	N/A
**Geographic location**							
	Rural and indigenous communities (n=124)	33 (26.6)	21 (16.9)	37 (29.8)	48 (38.7)	64 (51.6)	60 (48.4)
	Town or city (n=610)	195 (32)	128 (21)	134 (22)	159 (26.1)	275 (45.1)	336 (55.1)
	Urban center (n=313)	153 (48.9)	131 (41.9)	135 (43.1)	153 (48.9)	194 (62)	119 (38)
**Care setting**							
	Primary and community care (n=367)	165 (45)	128 (34.9)	136 (37.1)	139 (37.9)	202 (55)	165 (45)
	Other hospitals (n=397)	107 (27)	75 (18.9)	83 (20.9)	119 (30)	179 (45.1)	218 (54.9)
	Teaching hospital (n=176)	63 (35.8)	39 (22.1)	49 (27.8)	55 (31.3)	93 (52.8)	83 (47.2)
	Other nonhospital (government and others; n=107)	44 (41.1)	37 (34.6)	37 (34.6)	47 (43.9)	57 (53.3)	50 (46.7)
**Patient record–keeping system**							
	Paper only (n=149)	39 (26.2)	34 (22.8)	33 (22.1)	31 (20.8)	54 (36.2)	95 (63.8)
	Fully electronic (n=280)	101 (36.1)	70 (25)	81 (28.9)	101 (36.1)	146 (52.1)	134 (47.9)
	Hybrid (n=618)	260 (42.1)	210 (34)	198 (32)	235 (38)	346 (56)	272 (44)
**Main care setting has a policy about the use of email to communicate with patient**							
	Yes	427 (40.8)	N/A	N/A	N/A	N/A	N/A

^a^Source: 2020 National Survey of Canadian Nurses, Canada Health Infoway, and Léger [5,27].

^b^RPM: remote patient monitoring.

^c^N/A: not applicable.

#### Age and Work Experience

Nurses virtually delivering care were aged, on average, 42 (SD 12.3) years, slightly half a year younger than the overall sample collected in 2020 (mean 42.5, SD 12.1 years). The aging of the nursing workforce is an important issue as it may lead to a nursing shortage [25], which would be detrimental for the Canadian health care system as it strives to respond to the growing demand for long-term care stemming from an aging population [18]. On average, nurses who had used telehealth tended to be younger (mean 38.7, SD 10.4 years) than their peers using the other virtual care modalities (mean 39.5, SD 11.0; 39.6, SD 11.2; and 40.58, SD 11.7 years for telemonitoring, videoconferencing, and secure email, respectively). Similarly, these nurses providing care through telehealth had, on average, fewer years of practice (mean 11.5, SD 10.3 years) than others using the other virtual care modalities (mean 12.3, SD 10.8; 12.8, SD 11.7; and 13.4, SD 11.1) years for videoconferencing, telemonitoring, and secure email, respectively). Nurses who lacked access to virtual care technologies, on average, were older (42.9, SD 11.9 years) and had more years of service (16.7, SD 11.8 years) than their counterparts accessing virtual care technologies.

#### Nursing Designation

As shown in Table 2, NPs constituted the nursing professionals who were the most likely to deliver care virtually in 2020 (135/159, 84.9%) regardless of the type of virtual care technology considered. Although RNs made up 68.34% (300,669/439,975) of the total supply of Canadian-regulated nurses and 69.08% (273,617/396,085) of the nurses employed in nursing-specific jobs in 2019 [18], they were more likely to report a lack of access to virtual care technologies (403/683, 59%) than other nursing designations. Given their role in the delivery of care across the care continuum, from primary care to end of life, RNs are well-positioned to bring about health transformations that benefit the Canadian health care system [28]. Therefore, having greater access to virtual care technologies can potentially optimize their scope of practice.

#### Skills and Knowledge to Use Virtual Care Technologies

Several studies have argued that nurses’ skills and knowledge are among the factors that facilitate the integration of virtual care into nursing practice [7-10,12]. The results in Table 3 show that more than half of the nurses who had used videoconferencing or telemonitoring in the past (232/407, 57% and 243/407, 59.7%, respectively) reported that they had the skills and knowledge to deliver care through videoconferencing and telemonitoring.

### Main Care Settings of Nurses Using Virtual Care Technologies

#### Overview

Table 3 shows some variations in the use of virtual care technologies by nurses across care settings. Primary and community care settings include nursing homes, long-term care facilities, and homecare, whereas other hospital settings embody community hospitals and continuing care or rehabilitation hospitals. Overall, nurses working in primary and community care settings were more likely to use virtual health services (202/367, 55%) than nurses working in other settings. In addition, the use of secure messaging (165/367, 44.9%) and telehealth (136/367, 37.1%) was more prevalent among primary and community care nurses. These results align with those of Taylor et al [7], who found disparities in frontline staff acceptance of telehealth within and across service settings.

#### Geographic Location of Main Care Setting

At their inception, virtual care models were intended to deliver care to rural communities with limited access to traditional health care facilities [29]. In early 2020, a few weeks before the pandemic, more nurses working in rural and remote communities were delivering care virtually to their patients than 3 years ago. Approximately 51.6% (64/124) of nurses serving rural and indigenous communities reported that they had used at least one of the virtual care technologies considered in the analysis, an increase from 30% in 2017 [5]. However, Table 3 shows that rural nurses were less likely to use secure messaging (33/124, 26.6%) and videoconferencing (21/124, 16.9%) relative to their town and urban peers. In addition, the growth in the proportion of nurses using virtual health has been more substantial for nurses in cities and urban centers than for nurses serving rural and indigenous communities [5]. Results of the 2020 National Survey of Canadian Nurses also suggested that nurses serving rural and indigenous communities were more likely to report a lack of skills (16/48, 33.3%) in using virtual care technologies relative to their peers serving in towns or cities (52/191, 27.2%) and urban centers (35/166, 21.1%). Similarly, lack of knowledge to interact with virtual care tools was more prevalent among rural nurses (15/49, 30.6%) than among nurses practicing in towns or cities (47/191, 24.6%) and urban centers (32/166, 19.3%). These disparities in competencies required to use virtual care could be one of the drivers of the discrepancies observed in the use of virtual care technologies between rural and urban nurses.

### Drivers of the Uptake of Virtual Care Technologies by Nurses

#### Overall Factors

Table 4 reports the results of the regression model specified earlier in equation 4. Overall, the model is statistically significant (*χ^2^*_14_=160.7; *P*<.001). Hence, the professional characteristics of the nurses, their perception, and the characteristics of the care setting where they practice have a joint effect on the probability that these nurses deliver care virtually. In terms of goodness of fit, the estimated model has allowed to correctly predict >60% (count *R^2^*=0.6693) of the probabilities for nurses to use virtual care or not.

Regarding the individual effect of each factor entering the model, of all the care setting–level factors considered, only 1 was found to have a significant effect on the probability for nurses to deliver care virtually. Nursing designation (*P*<.001), perceived quality of care delivered by the care setting (*P*<.001), and type of patient record–keeping system (*P*=.04) all had a statistically significant effect on the probability of nurses using virtual care when their categories entering the model were taken together. In contrast, the model failed to provide statistical evidence for the effect of work experience, type of care setting, and geographic location of the care setting on predicting whether a nurse used virtually health. Therefore, knowing where a nurse worked, either their health care setting or the location, may not contribute to predicting whether they delivered care virtually or not.

For nursing designation, some differences across the considered designations are worth noting. NPs and the combined group of LPNs, RPNs, and CNSs were respectively 7 times (odds ratio 7.04) and more than twice (odds ratio 2.24) as likely to use virtual care technologies than RNs. These results confirm the disparities found earlier in the use of virtual care by nurses across designations. The data showed that the proportions of NPs and the combined group of LPNs, RPNs, and CNSs delivering care virtually in 2020 outnumbered the proportions of RNs. Similarly, there were some discrepancies in the probability of using virtual care across the perception of overall quality of care in the care setting. Indeed, nurses who perceived the overall quality of care in their care setting as excellent or good were more than twice (odds ratios 2.14 and 2.04, respectively) as likely to use virtual care relative to those who perceived the quality as poor. Nurses who felt that the overall quality of care in their care setting was poor did not significantly differ from those who rated the quality as fair in terms of their probability of using virtual health.

**Table 4:** Results of the estimation of model defined in equation 4^a^.

Predictors		Log-odds coefficient	SE	Odds ratio (95% CI)	Average marginal effect	*P* value >|z|	
**Nursing designation**		N/A^b^	N/A	N/A	N/A	<.001^c^	
	RN^d,e^	N/A	N/A	N/A	N/A	N/A	
	NP^f^	1.951^g^	0.248	7.04 (4.33-11.43)	0.417	<.001	
	LPN^h^, RPN^i^, and CNS^j,k^	0.805^g^	0.196	2.24 (1.52-3.28)	0.187	<.001	
	Other	0.218	0.285	1.24 (0.71-2.18)	0.049	.45	
Work experience		–0.001	0.006	1.00 (0.98-1.01)	–0.0001	.93	
**Perceived quality of care delivered by setting**		N/A	N/A	N/A	N/A	<.001^c^	
	Poor^e^	N/A	N/A	N/A	N/A	N/A	
	Fair	0.021	0.281	1.02 (0.59-1.77)	0.004	.94	
	Good	0.711^g^	0.251	2.04 (1.24-3.33)	0.153	.005	
	Excellent	0.763^g^	0.256	2.14 (1.29-3.55)	0.165	.003	
**Care setting**							.34^c^
	Primary and community care^e^	N/A	N/A	N/A	N/A	N/A	
	Other hospitals	–0.248	0.161	0.78 (0.57-1.07)	–0.052	.12	
	Teaching hospital	–0.006	0.205	0.99 (0.67-1.49)	–0.001	.98	
	Other nonhospital	0.052	0.241	1.05 (0.66-1.69)	0.011	.83	
**Patient record–keeping system**		N/A	N/A	N/A	N/A	.04^c^	
	Paper^e^	N/A	N/A	N/A	N/A	N/A	
	Fully electronic	0.507^l^	0.232	1.66 (1.11-2.49)	0.105	.03	
	Hybrid	0.511^l^	0.206	1.67 (1.05-2.62)	0.106	.01	
**Geographic location of care setting**		N/A	N/A	N/A	N/A	.13^c^	
	Rural and indigenous communities^e^	N/A	N/A	N/A	N/A	N/A	
	Town or city	–0.267	0.213	0.77 (0.51-1.16)	–0.057	.21	
	Urban center	0.027	0.238	1.03 (0.64-1.64)	0.006	.91	
	Constant	–1.078^g^	0.350	N/A	N/A	.002	

^a^Number of observations=1131; log likelihood –480.386; Akaike Information Criteria 990.772; number of iterations=4; likelihood ratio *χ^2^*_14_=160.2; probability>*χ^2^*=0.0; pseudo *R^2^* (McFadden)=0.3722; count *R^2^*=0.6693.

^b^N/A: not applicable.

^c^To test for the overall effect of each categorical variable on the dependent variable (probability to use virtual care), we used a Wald test. These values correspond to the *P* value associated with the chi-square statistic calculated for the Wald test for each categorical variable.

^d^RN: registered nurse.

^e^Omitted category of the categorical variable entering the model to avoid the problem of multicollinearity.

^f^NP: nurse practitioner.

^g^*P*<.01.

^h^LPN: licensed practical nurse.

^i^RPN: registered psychiatric nurse.

^j^CNS: clinical nurse specialist.

^k^To deal with the low sample sizes for LPNs, RPNs, and CNSs, we grouped them in a single category. We recognize that these 3 nursing professionals differ significantly in terms of their education, duties, and work settings.

^l^*P*<.05.

The patient record–keeping system that nurses had access to was found to have a statistically significant global effect on the probability of using virtual care. However, nurses with access to fully electronic and hybrid record–keeping systems were approximately twice as likely to deliver care virtually (odds ratios 1.66 and 1.67, respectively) than their peers using paper charts.

Although it was found to have no statistically significant effect on the probability of using virtual care, work experience had a negative effect on the outcome variable of the model. This indicates that nurses with more years of nursing practice would be less likely to adopt virtual technologies in clinical care. However, the marginal effect of work experience on the probability of using virtual care was found to be minimal.

#### Drivers Related to Virtual Care Through Secure Email and Videoconference

A policy-related variable was collected for nurses who delivered care through secure email, whereas skills and knowledge data were gathered for those delivering care through videoconferencing technology. A chi-square test of independence was conducted to investigate whether these factors were associated with the use of virtual care for these 2 technologies. The results of the test are provided in Table 5 and concern the subsample of nurses delivering care through secure email and videoconferencing. Table 5 shows that the existence of a policy on the use of email to securely communicate with patients in the care setting was statistically weakly associated with the use of email by nurses to consult with their patients (*χ^2^*_2_=61.4; *P*<.001; Cramer V=0.24). For nurses who had consulted directly with a patient via videoconference, their perception of the quality of care delivered through these virtual technologies was statistically driven by their skills and knowledge (*χ^2^*_4_=308.7, *P*<.001; and *χ^2^*_4_=283.4, *P*<.001, respectively). Furthermore, these self-assessment factors were strongly associated with perceived quality of care (Cramer V=0.62 and Cramer V=0.59, respectively). Therefore, taking into consideration how nurses feel about their skills and knowledge to use videoconference can help predict their perception of the quality of care delivered through these digital means. Consequently, this will improve their adoption of these virtual technologies.

**Table 5:** Independence test and association measures.

Characteristics and independent variables			Dependent variables		Chi-square (*df*)		*P* value (2-sided)		Level of association^a^
**Policy**									
	Policy about the use of email to communicate with the patient	Had used secure email to deliver care		61.4 (2)		<.001		0.24	
**Perception**									
	Skills to use virtual care	Perception of quality of care delivered through videoconference or telemonitoring		308.7 (4)		<.001		0.62	
	Knowledge to use virtual care	Perception of quality of care delivered through videoconference or telemonitoring		283.4 (4)		<.001		0.59	

^a^Cramer V was calculated for the level of association.

## Discussion

### Principal Findings

Our study endeavored to quantify the effect of some of the drivers that were found to affect the adoption of virtual care in nursing practice by a spate of previous researchers who relied on qualitative methods. We were able to quantify the distribution of the uptake of virtual care across nursing designation, care setting, and geographic region. For instance, the results suggested that RNs and rural nurses reported the lowest adoption rate for the use of virtual care. For nursing designations, we found statistical evidence for disparities in the use of virtual care across designations. This could be linked to the differences in the scope of practice, roles, and competencies across all levels of nursing practice. For geographic location, Chen et al [24] found significant disparities in the adoption of telehealth between rural and urban hospitals. In addition to allowing them to reach out to patients in remote sites, virtual care is regarded by rural nurses as a mechanism that offers them the possibility to enhance and maintain skills and professional knowledge [9,30]. Moreover, some scholars have argued that telehealth could positively affect rural clinicians’ occupational well-being, leading to greater retention and recruitment rates in remote care settings [31]. In addition, the results expounded in this paper shed light on the modalities of virtual care technologies that are being used across care settings and geographic locations. This will enable decision-makers to pinpoint the gaps in terms of the use of virtual health technologies. Thus, the allocation of digital health technology resources in rural and urban settings could improve.

The results of our regression model provided statistical evidence for the collective effect of nurses’ professional characteristics, their perceptions, and the characteristics of the care settings where they practice their use of virtual care technologies. All these factors entering our regression model had already been identified in the literature. However, previous studies have looked into them separately but not as a whole. Furthermore, these studies failed to report on the magnitude of the effect of these factors on the use of virtual care in nursing care. Our results suggest that the effect of work experience on the use of virtual care was very marginal, signaling that this factor would not be a relevant predictor of the adoption of virtual care in nursing practice. Similarly, the existence of a policy on the use of email to communicate with patients in the care setting was found to be weakly associated with the use of email. Nurses have an instrumental role to play in supporting the design of policies and regulations aimed at supporting virtual care [32].

The significant association between the patient record–keeping system and the use of virtual care technologies is a promising finding that will help to better inform initiatives aimed at achieving interoperability. Our results suggest that nurses who access electronic EMRs have a higher likelihood of delivering care virtually. Earlier studies found that a lack of interoperability could fragilize the uptake of virtual care technologies by frontline workers [7].

Previous studies were limited to identifying enablers and inhibitors; however, they provided us with the main elements for our investigation. Therefore, by filling the gaps in terms of quantitative analysis of the drivers of virtual care adoption by nurses, our study complements these previous studies. However, similar to the study by Mair et al [13], we still believe that the integration of virtual care technologies into nursing practice can be convoluted and multifaceted. Concurrently, virtual care integration will substantially benefit nurses as the scope of their practice will be more developed, and nursing care delivery will become more flexible [6].

### Limitations

Our study used data generated from the 2020 National Survey of Canadian Nurses. This survey combined the CNA and CNIA membership lists to establish the survey sample. In addition, other strategies were implemented to mitigate the lack of coverage and completeness resulting from the conflation of CNA and CNIA membership lists. Despite these strategies, nonmembers and underrepresented groups of nurses might have been left out from our analysis. Future studies could endeavor to obtain a more complete list of the Canadian-regulated nursing workforce from the CIHI. Moreover, limited professional characteristics and care setting–level factors were collected through the survey. In addition, factors related to the types of patients served by nurses were omitted from our analysis, although they were highlighted as relevant drivers of adoption in the literature. Consequently, several pertinent explanatory variables might have been omitted from the regression model. This omission of relevant predictors could generate some specification errors, although the predictive power of the model was satisfactory, and its overall significance passed the statistical hypothesis test. Nevertheless, our study laid the foundation for future research aiming to undertake a quantitative analysis of the drivers of virtual care adoption in nursing practice. From a resource allocation standpoint, future research could investigate the factors that drive the number of visits delivered by nurses through virtual care means and how these factors vary across virtual care technologies.

### Conclusions

Our investigations suggest that the use of virtual care in nursing practice is mostly driven by three factors: (1) nursing designation, (2) the geographic location where a nurse practices, and (3) the type of patient record–keeping system accessed in the care setting. The disparities observed in the use of virtual care across nursing designations should draw the attention of both nursing leaders in care settings and nursing educators. These leaders need to ensure that virtual care technologies are accessible to nurses at all levels of practice and digital training is well embedded into nursing education programs. On the geographic location front, the disparities observed could be alleviated through the balanced and effective allocation of digital health resources between urban and rural regions. Finally, the association between access to EMRs and the use of virtual care by nurses should foster the adoption of more modernized patient record–keeping systems. This will have some positive implications for interoperability and health care delivery.

## Appendix

Multimedia Appendix 1 Increase in the use of virtual care in nursing care (December 2021).

## Abbreviations

CIHI: Canadian Institute for Health Information
CNA: Canadian Nurses Association
CNIA: Canadian Nursing Informatics Association
CNS: clinical nurse specialist
EMR: electronic medical record
LPN: licensed practical nurse
NP: nurse practitioner
RN: registered nurse
RPM: remote patient monitoring
RPN: registered psychiatric nurse
