# Correction: Uncovering Important Drivers of the Increase in the Use of Virtual Care Technologies in Nursing Care: Quantitative Analysis From the 2020 National Survey of Canadian Nurses

**DOI:** 10.2196/50791

**Published:** 2023-10-13

**Authors:** Waldo Beauséjour, Simon Hagens

**Affiliations:** 1 Canada Health Infoway Toronto, ON Canada

In “Uncovering Important Drivers of the Increase in the Use of Virtual Care Technologies in Nursing Care: Quantitative Analysis From the 2020 National Survey of Canadian Nurses” (JMIR Nursing 2022;5(1):e33586) the authors noted one correction.

In the published manuscript, the Acknowledgments read as follows:

The authors would like to extend their acknowledgments to the Performance Analytics team at Canada Health Infoway for reviewing this manuscript before submission.

The publication costs for this article have been covered by Canada Health Infoway Inc, a non-profit corporation funded by the Government of Canada.

They have now been changed to:

The authors would like to extend their acknowledgments to the Performance Analytics team at Canada Health Infoway for reviewing this manuscript before submission.

The authors would like to thank the following organizations and individuals who significantly contributed to the design, implementation, distribution, dissemination, and analysis of the results of the 2020 National Survey of Canadian Nurses. The data and results from this survey served as the main source for their study: The Canadian Nurses Association (CNA); The Canadian Nursing Informatics Association (CNIA); L’Association Québécoise des infirmières et infirmiers en systèmes et technologies de l’information (AQIISTI); L’Ordre des infirmières et infirmiers d Québec (OIIQ); Glynda Rees, RN, MSN, President, Canadian Nursing Informatics Association, Faculty, BCIT School of Nursing; Éric Maillet, RN, PhD, Assistant Professor at the School of Nursing and Director of Health Informatics Programs at the University of Sherbrooke; Leanne M. Currie, RN, PhD, Associate Professor at University of British Columbia School of Nursing; Peggy White, former CNIA president; and Josette Roussel, RN, MSc (Nursing), MEd, former Executive Advisor at the Canadian Nurses Association.

The publication costs for this article have been covered by Canada Health Infoway Inc, a non-profit corporation funded by the Government of Canada.

The correction will appear in the online version of the paper on the JMIR Publications website on October 13, 2023 together with the publication of this correction notice. Because this was made after submission to PubMed, PubMed Central, and other full-text repositories, the corrected article has also been resubmitted to those repositories.

