# Supplementary material for: Uncovering Important Drivers of the Increase in the Use of Virtual Care Technologies in Nursing Care: Quantitative Analysis From the 2020 National Survey of Canadian Nurses
Source: JMIR Nurs. 2022 Mar 31;5(1):e33586. doi: 10.2196/33586 (PMC9015777; doi:10.2196/33586)
Supplement: Multimedia Appendix 1 [file nursing_v5i1e33586_app1.docx]

**Appendix**

**Questions on access to virtual care technologies**

*An e-visit is where a patient initiates a consultation via secure e-mail to their health provider(s) about a specific health question or concern.*

1. In the past 3 months, how many times have you carried out an e-visit using secure e-mail to respond to a patient-initiated e-mail consultation about a specific health issue or concern? *If you have not done any in the past 3 months, please type in ‘0’.*

____ [NUMERIC ENTRY. RANGE 0-50]

- Do not recall

*A virtual visit occurs when a virtual video conference is conducted between a patient and a clinical provider. A virtual visit may be patient-initiated or coordinated by or between health facilities.*

1. **In the past 3 months, how many times have you:**

|  | **Number of times in the past 3 months** |
| --- | --- |
| Consulted directly with a patient via virtual videoconference | __ **[NUMERIC ENTRY. RANGE 0-50]** 🞎 Do not recall |
| Carried out a virtual visit while in-person with a patient with a remote clinical provider (e.g., telehealth) | ___ **[NUMERIC ENTRY. RANGE 0-50]** 🞎 Do not recall |

1. Does your MAIN Care setting have a policy about the use of e-mail to securely communicate with patients about their care?

- Yes
- No
- Don’t know

1. In the past 3 months, how many patients under your care have been enrolled in remote telemonitoring services (e.g. telehomecare)? *If you have not had any patients enroll in remote telemonitoring services in the past 3 months, please type in ‘0’.*

____ [NUMERIC ENTRY. RANGE 0-50]

- Do not recall

IF in Q44=”Consulted directly with a patient via virtual videoconference” is >0 OR DO NOT RECALL OR IF Q46 IS >0 OR DO NOT RECALL, ASK Q47.

1. As you have consulted directly with a patient via virtual videoconference or used remote telemonitoring services (e.g. telehomecare) in the last 3 months, to what degree do you agree or disagree with the following statements:

| Statement | Strongly Agree | Moderately Agree | Moderately Disagree | Strongly Disagree | Not Sure |
| --- | --- | --- | --- | --- | --- |
| I currently have the skills to use virtual videoconference and/or telemonitoring (e.g. telehomecare) in my nursing practice. | □ | □ | □ | □ | □ |
| I currently have the knowledge to use virtual videoconference and/or telemonitoring (e.g. telehomecare) in my nursing practice. | □ | □ | □ | □ | □ |
| I provide more efficient health care with virtual videoconference and/or telemonitoring (e.g. telehomecare) | □ | □ | □ | □ | □ |
